# Rural-urban difference in colorectal cancer mortality

**DOI:** 10.7150/jca.59434

**Published:** 2021-04-13

**Authors:** Tomoya Urakawa, Akihiro Saitsu, Jun Watanabe, Kazuhiko Kotani

**Affiliations:** Division of Community and Family Medicine, Center for Community Medicine, Jichi Medical University, Shimotsuke-City, Tochigi, Japan.

We read with great interest the recent study by Daniel et al., which showed a different response to knowledge and practice in relation to colorectal cancer screening between minority and majority populations in the deep south of the U.S. 1. As Daniel et al. stated 1, rurality is a possible contributor to increased mortality in patients with colorectal cancer because several factors related to lifestyle, and socioeconomics and healthcare systems are associated with the diagnosis and clinical course of this disease. Thus, the study assumed the difference in responses to colorectal cancer screening between rural and urban residents - interestingly, however, few differences were found 1. This might be an unexpected finding, as it is generally considered that rural-urban health inequity in cancer treatment is an issue that remains to be resolved.

Actually, what is the worldwide status of the rural-urban difference in colorectal cancer mortality, which is an ultimate outcome of screening? We performed a search of the PubMed database to identify original articles published prior to December 2020, with a population-based cohort design that reported the colorectal cancer mortality between rural and urban areas. The keywords were “rural[All Fields] OR urban[All Fields]) AND (“rectal neoplasms”[MeSH Terms] OR (“rectal”[All Fields] AND “neoplasms”[All Fields]) OR “rectal neoplasms”[All Fields] OR (“rectum”[All Fields] AND “cancer”[All Fields]) OR “rectum cancer”[All Fields]) AND (“mortality”[Subheading] OR “mortality”[All Fields] OR “mortality”[MeSH Terms]) AND English[lang]. We obtained the hazard ratio or relative risk for cancer death between rural and urban areas, with adjustment for various co-variates.

In total, 143 English language articles were identified using the keywords. After evaluating the full text, five articles were finally eligible for the present review (Table 1) 2-6. As a result, one study reported that the mortality risk was high in urban areas 2, while two studies reported that the mortality risk was high in rural areas 3, 5. The remaining two studies reported no clear difference in risk between rural and urban areas 4, 6.

The results of the present review were therefore controversial; namely, a rural-urban difference in colorectal cancer mortality was not obviously proven. Of note, the present findings appear to be in line with the results reported by Daniel et al. 1. While Daniel et al. hypothesized that rural residents often acknowledge a family history of cancer and cope with it well, more studies are warranted to clarify the detailed reasons for these findings.

## Figures and Tables

**Table 1:** The hazard ratio or relative risk of colorectal cancer deaths between rural and urban areas

Study/Country [reference No.]	Cohort duration	Population (number)	Age (number)	Cancer type	Residence	Hazard ratio/relative risk (95% confidence interval)	Adjusted co-variables
Kassim, 2019/ China 6	2007-2011	Male 143 Female 91	54.96 years	Right colon	UrbanRural	1.0 (reference) 1.082 (0.765-1.529)	Age, gender, smoking, drinking, residence, cancer grade, cancer stage, chemotherapy
		Male 147Female 94	53.93 years	Left colon	UrbanRural	1.0 (reference) 1.151 (0.666-1.988)	
		Male 369Female 234	56.86 years	Rectum	UrbanRural	1.0 (reference) 0.934 (0.752-1.159)	
Feller, 2018/ Swiss 5	2000-2008	Male 5,700Female 4,388	< 50 years (668) 50-64 years (3,007) 65-74 years (3,180) 75-84 years (3,233)	Colorectum	Urban Rural	1.0 (reference) 1.15 (1.02-1.30)	Age, civil status, nationality, urbanity, residence, cancer localization, cancer stage, socioeconomic position
Hines, 2014/ United States 4	2000-2012	Male 10,702Female 9,742	45-64 years (9,675) 65-74 years (5,890) 75-85 years (4,879)	Colorectum	Urban Rural	1.0 (reference) 1.02 (0.94-1.12)	Age, gender, race, cancer stage, cancer grade, geography, treatment (surgery, chemotherapy or radiation), socioeconomic status
Hines, 2012/ United States 3	1992-2007	Male 7,365Female 7,809	Rural 68.4 years Urban 65.8 years	Colon Rectum	Urban Rural Urban Rural	1.0 (reference) 1.15 (1.01-1.32) 1.0 (reference) 1.13 (0.91-1.41)	Age, gender, cancer stage, cancer grade, treatment (surgery or radiation)
Vassallo, 1994/ Uruguay 2	1988-1992	Male 1,121,250Female 1,209,700	< 55 years (1,664,150) 55-64 years (316,450) 65-74 years (209,500) >75 years (140,850)	Colon	Urban Rural	Male 1.50 (1.32-1.70)Female 1.17 (0.34-1.34) Male 1.0 (reference) Female 1.0 (reference)	Age, residence
				Rectum	Urban Rural	Male 1.89 (1.51-2.35)Female 1.35 (1.06-1.72) Male 1.0 (reference) Female 1.0 (reference)	
